# Diagnostic performance of liver stiffness as marker of liver involvement in systemic immunoglobulin light chain (AL) amyloidosis

**DOI:** 10.1007/s00277-024-05932-4

**Published:** 2024-08-16

**Authors:** Anne F. Brunger, Hendrea S.A. Tingen, Johan Bijzet, Ronald van Rheenen, Hans Blokzijl, Wilfried W. H. Roeloffzen, Ewout J. Houwerzijl, Friso L. H. Muntinghe, Riemer H. J. A. Slart, Reinold O. B. Gans, Christoph Kimmich, Bouke P. C. Hazenberg, Hans L. A. Nienhuis

**Affiliations:** 1 Departments of Rheumatology and Clinical Immunology, Amyloidosis Center of Expertise, University Medical Center Groningen, Groningen, The Netherlands; 2 Departments of Nuclear Medicine and Molecular Imaging, Amyloidosis Center of Expertise, University Medical Center Groningen, Groningen, The Netherlands; 3 Departments of Laboratory medicine, Amyloidosis Center of Expertise, University Medical Center Groningen, Groningen, The Netherlands; 4 Departments of Gastroenterology, Amyloidosis Center of Expertise, University Medical Center Groningen, Groningen, The Netherlands; 5 Departments of Hematology, Amyloidosis Center of Expertise, University Medical Center Groningen, Groningen, The Netherlands; 6 Departments of Internal Medicine, Amyloidosis Center of Expertise, University Medical Center Groningen, Groningen, The Netherlands; 7 Department of Nuclear Medicine, Medical Center Leeuwarden, Leeuwarden, The Netherlands; 8 Department of Oncology and Hematology, Klinikum Oldenburg, University Medicine Oldenburg, Oldenburg, Germany

**Keywords:** AL amyloidosis, Fibroscan, Hepatic amyloidosis, Liver stiffness, SAP scintigraphy

## Abstract

Objective: To investigate the diagnostic performance of liver stiffness for detecting liver involvement in immunoglobulin light chain (AL) amyloidosis. Methods: Liver stiffness was measured using transient elastography in 71 patients with systemic AL amyloidosis and 18 patients with wild type transthyretin (ATTRwt) amyloidosis with cardiomyopathy. Both non-invasive consensus criteria and serum amyloid P component (SAP) scintigraphy were used as substitute standards instead of liver biopsy for establishing liver involvement. Results: Liver stiffness was higher in AL amyloidosis patients with liver involvement than in those without: this was observed using both consensus criteria (median 14.4 kPa vs. 8.1 kPa; *p* = 0.001) and SAP scintigraphy (median 20.9 kPa vs. 6.2 kPa; *p* < 0.001). Liver stiffness was also higher in AL amyloidosis patients with liver involvement compared to AL and ATTRwt amyloidosis patients with cardiac involvement. Based on receiver operating characteristic (ROC) curves a cut-off value of 14.4 kPa for stiffness was optimal to indicate liver involvement, providing sensitivity and specificity of 50% and 74%, respectively, using the consensus criteria and 63% and 90%, respectively, using SAP scintigraphy as standard. Conclusion: Liver stiffness is a promising tool to establish liver involvement in AL amyloidosis having potential to become part of updated criteria for liver involvement.

**Supplementary Information:**

The online version contains supplementary material available at 10.1007/s00277-024-05932-4.

## Introduction

In systemic immunoglobulin light chain (AL) amyloidosis, heart involvement has most impact on survival [1]. However, liver involvement also affects prognosis [2, 3] and increases the risk of treatment-related toxicity [4]. Identification of liver involvement at the start of treatment provides parameters by which to monitor treatment response.

Amyloid fibrils are insoluble fibers that aggregate in the extracellular space of organs consequently stiffening the organs [5]. That amyloid deposition causes stiffening of organs has been recognized for centuries [6]. Stiffness of the liver can easily and non-invasively be measured with transient elastography. The procedure is widely available, quick, provides immediate results, is reproducible, is not difficult to learn and has an excellent inter- and intra-observer agreement in patients with chronic liver disease [7, 8]. Loustaud-Ratti et al. investigated the diagnostic value of liver stiffness for liver involvement in 41 AL amyloidosis patients and concluded that liver involvement is very likely in an AL amyloidosis patient with an elastography value of ≥ 17.3 kPa [9].

A liver biopsy showing interstitial deposits of amyloid is regarded as the gold standard for liver involvement [10]. Validating the diagnostic performance of liver stiffness for liver involvement in amyloidosis against the liver biopsy as gold standard, however, exposes participants to unjustifiable safety risks such as a bleeding risk of about 2% [2, 11]. Therefore, two substitute standards for liver involvement are more suitable to be used instead.

In 2005 criteria for organ involvement and treatment response were formulated by a consensus panel comprising 13 specialists actively involved in the treatment of patients with amyloidosis [10]. Liver involvement is defined as implicated when amyloid is diagnosed at another site in a patient with either hepatomegaly (defined as total craniocaudal liver span greater than 15 cm in the absence of heart failure) or when the serum alkaline phosphatase (ALP) value is above 1.5 times the upper limit of the institutional normal value. These non-invasive consensus criteria are generally used as substitute standard for liver involvement. A disadvantage is that these non-invasive consensus criteria have never been validated systematically against the liver biopsy.

In 1988 ^123^iodine-labeled serum amyloid P component (SAP) scintigraphy was introduced as non-invasive method to detect amyloid deposition in organs including liver, kidneys, adrenals, and spleen [12, 13]. SAP scintigraphy is a sensitive and specific method to detect involvement of organs such as the liver [14]. As such it can be regarded as second substitute standard for liver involvement. Though SAP scintigraphy has not systematically been validated against the liver biopsy, subgroup analysis showed promising results in the study of Lovat et al. [15]. In this study a liver biopsy was available in a subgroup comprising 59 of the 484 patients, showing 100% concordance between hepatic SAP scintigraphy and the presence or absence of parenchymal amyloid deposits on liver histology.

In clinical studies, the reported prevalence of liver involvement in AL amyloidosis ranges from 8 to 54% [15–19]. The wide range can partly be explained by the way liver involvement was assessed in these studies. Autopsy studies suggest that in far-advanced disease amyloid is present in the liver of most patients [20]. Identification of liver involvement in AL amyloidosis during life is mostly based on the two substitute standards, usually on the non-invasive consensus criteria and occasionally on SAP scintigraphy [21].

The aim of this study was to investigate the diagnostic performance of liver stiffness for detecting liver involvement in AL amyloidosis as defined by the non-invasive consensus criteria [10], hereafter condensed as consensus criteria, and by SAP scintigraphy.

## Methods

### Study population

Treatment-naive patients with AL amyloidosis referred for evaluation at the Amyloidosis Center of Expertise of the University Medical Center Groningen (UMCG) between March 2016 and March 2022 who had undergone both transient elastography and SAP scintigraphy and who provided verbal informed consent were included. More than half of the AL amyloidosis patients were admitted to the hospital for clinical evaluation and consecutively included. The other patients visited the outpatient clinic and underwent a fibroscan because of the suspicion of liver involvement. Additionally, patients with wild type ATTR amyloidosis with cardiomyopathy (ATTRwt-CM) were included in this study as cardiac amyloidosis controls after verbal informed consent was obtained. Patients were diagnosed according to current guidelines [22, 23].

The diagnostic performance of liver stiffness was cross-sectionally assessed. The following clinical and biological parameters were recorded at the time of the liver stiffness measurements׃ age, sex, body mass index (BMI), serum levels of creatinine, alanine aminotransferase (ALT), alkaline phosphatase (ALP), gamma-glutamyl transferase (GGT), Troponin T, N-terminal pro B-type natriuretic peptide (NT-proBNP), and urine analysis on proteinuria. Liver involvement was assessed with both the consensus criteria as proposed by the consensus panel during the 10th International Symposium on Amyloid and Amyloidosis in 2004 (total craniocaudal liver span larger than 15 cm in the absence of heart failure and/or ALP value above 1.5 times the upper limit of the institutional reference values) [10] and SAP scintigraphy. SAP scintigraphy was performed at the time of liver stiffness measurement or at least within 2 months. The institutional upper reference limit of ALP was 98 IU/L for women and 115 IU/L for men, therefore > 147 IU/L for women and > 172 IU/L for men was indicative of liver involvement. For determining the liver span the craniocaudal liver size was measured on the CT images provided by SAP scintigraphy [21]. Other causes of chronic liver diseases (e.g. viral hepatitis, metabolic dysfunction associated steatotic liver disease) were ruled out on indication. Heart failure was defined as signs of fluid overload (increased central venous pressure, peripheral oedema or basal crepitation in the lungs) in a patient with cardiac involvement. Furthermore, electrocardiogram, echocardiography and on indication cardiac Magnetic Resonance Imaging were performed to evaluate cardiac involvement. Cardiac involvement was defined according to the European Society of Cardiology (ESC) working group position statement of diagnosis and treatment of cardiac amyloidosis [23].

The patients with AL amyloidosis were divided into three subgroups i.e., patients with liver involvement (established by using either the consensus criteria or the SAP scintigraphy as substitute standard), patients with heart involvement without liver involvement, and patients with neither heart nor liver involvement. The ATTRwt-CM amyloidosis patients were regarded as pure cardiac amyloidosis disease controls because liver involvement is not a disease manifestation of this type of amyloidosis.

Additionally, liver stiffness measurements were obtained in 8 AL amyloidosis patients with liver involvement (as determined by SAP scintigraphy) before the start of treatment and after at least 6 months of treatment.

All procedures were in compliance with the Declaration of Helsinki. The study was approved by the medical ethical committee of the University Medical Center Groningen (registration number: 17471).

### SAP scintigraphy

The SAP scintigraphy procedure was performed as described elsewhere [24]. The images were visually scored in a semi-quantitative way by comparing organ uptake directly or indirectly to the normal blood-pool distribution [25]. Organ involvement was graded 3+ (overwhelming uptake), 2+ (intense uptake), 1+ (positive uptake without any doubt), ± (weak or doubtful), and 0 (normal) [26]. For establishing liver involvement, the liver uptake on the anterior view is compared with the normal blood-pool in the heart. In healthy individuals there is no organ deposition of [^123^ I]-SAP and the tracer is then confined to the blood pool in the circulation and in major organs [13, 27, 28]. A quantitative organ to blood pool ratio was used to define organ involvement with high specificity: a liver to blood pool ratio (LBR) > 1.0 was indicative for liver involvement [29]. LBR, a continuous variable, was used as marker of liver amyloid load detected by SAP scintigraphy.

### Transient elastography

Transient elastography (Fibroscan, Echosens, Paris, France) was used for measuring liver stiffness. Measurements were made on the right liver lobe, through the intercostal space, with the patient in supine position with the right arm behind the head to enlarge the intercostal space. The tip of the probe transducer was placed on the skin between the rib bones at the level of the right liver lobe. The operator, assisted by a time motion ultrasound image, located a liver portion at least 6 cm thick and free of large vascular structures. The operator then pressed the probe button to begin the measurements. The median liver stiffness was calculated and expressed in kilopascal (kPa). An XL probe was used for obese patients. The manufacturer recommends caution with the interpretation of results of < 10 valid measurements and/or an interquartile range/median ratio (IQR/M) > 0.30 [7]. We therefore evaluated differences in liver stiffness measurements in 11 AL amyloidosis patients in whom transient elastography was performed a second time because the first measurement did not meet these recommendations. No differences were found between results that did or did not meet the recommendations (Supplementary Figure S1). Subsequently groups were made including and excluding measurements that did not meet these recommendations. When no differences in outcomes were found between these groups all data were used for further analysis. All measurements were performed by the same operator (HN).

### Statistical analysis

The Kruskal-Wallis test followed by the Games-Howell post hoc analysis was used to compare liver stiffness measurements and cardiac characteristics among different groups. Univariate correlations were assessed with Spearman’s correlations coefficient (r_s_). Multiple variate linear regression analysis with the enter method of inclusion was performed using liver stiffness as dependent variable and consensus criteria and SAP scintigraphy as independent variables. Receiver operating characteristic (ROC) curves were constructed to assess the diagnostic accuracy of liver stiffness in comparison to liver tracer uptake (LBR) on [^123^I]-SAP scan with consensus criteria as standard for liver involvement and to assess the diagnostic accuracy of liver stiffness in comparison to ALP and liver span with SAP scintigraphy as standard for liver involvement. Except when stated otherwise, values are expressed as mean ± standard deviation when normally distributed and as median (interquartile range) when non-normally distributed. A p-value ≤ 0.05 was considered significant. Statistical analyses were performed using SPSS version 23 (IBM Corp, Armonk, New York, USA) and Prism GraphPad version 8 (GraphPad Software, Inc, La Jolla, CA, USA).

## Results

### Patients and clinical characteristics

Seventy-one treatment-naïve AL amyloidosis patients were evaluated with both transient elastography and SAP scintigraphy as part of their clinical work-up: 40 consecutive patients who were admitted to the hospital and 31 patients of the outpatient clinic with suspicion of liver involvement. In addition, 18 ATTRwt-CM patients were included as cardiac amyloidosis controls. Characteristics are shown in Table 1. None of the patients included in this study used more than 2 units of alcohol per day. The test results of all individual patients can be found in Supplementary Figure S2. In three patients, abnormal liver enzyme levels were among the presenting features. After ruling out the usual causes of chronic liver disease, they underwent liver biopsies. All three biopsies showed interstitial amyloid deposits, and all three patients had liver involvement according to both standards.

**Table 1:** Characteristics of AL amyloidosis patients and ATTRwt-CM controls

	AL amyloidosis patients *N* = 71	ATTRwt-CM controls *N* = 18
Characteristics	Mean (SD), median (IQR)^#^	Mean (SD), median (IQR)^#^
**Gender**,** female/male**	24/47	0/18
**Age (years)**	66 (9)	76 (5)
**BMI (kg/m** ^**2**^ **)**	24 (4.1)	25 (3.1)
**Alanine aminotransferase (IU/L)**	25 (16–37)	24 (17–29)
**Alkaline phosphatase (IU/L)**	88 (70–135)	91 (76–120)
**Gamma-glutamyl transferase (IU/L)**	60 (23–221)	64 (39–140)
**NT-proBNP (pg/ml)**	1969 (394–5212)	2395 (1496–3744)
**Troponin T (ng/L)**	46 (19–98)	65 (46–84)
**Mean left ventricular wall thickness (mm)**	12 (11–15)	18 (14–21)
**Serum creatinine (µmol/L)**	101 (78–136)	93 (82–119)
**Proteinuria (g/24 hours)**	0.6 (0.1–6.9)	0.0 (0-0.1)
**Liver stiffness (kPa)**	9.9 (5.9–26.6)	9.7 (6.3–14.4)
**Tracer uptake (LBR) on [** ^**123**^ **I]-SAP scan**	1.1 (0.9–2.2)	N.A.
**Liver span (cm)**	17.5 (15–20)	N.A.

BMI, body mass index; IU/L: international units per liter; NT-proBNP, N-terminal pro B-type natriuretic peptide; kPa: kilopascal; LBR, liver-blood ratio; [^123^I]-SAP scan, iodine-123 labeled serum amyloid P component scintigraphy; NA, not applicable. #Statistics: data are expressed as median (interquartile range, from 25th percentile to 75th percentile, IQR), or mean (standard deviation, SD) where appropriate

**Table 2:** Liver stiffness values in different AL amyloidosis subgroups and ATTRwt-CM group A: liver involvement based on consensus criteria

**A: Liver involvement based on consensus criteria**					
**Type**	**Organ involvement**	**Number of treatment-naive patients**	**Median liver stiffness in kPa (IQR)**	Number of patients in whom liver stiffness met quality criteria*	**Median liver stiffness in kPa (IQR)**
AL	Liver	44	14.4 (6.6–56.4)	36	15.9 (6.5–70.3)
AL	Heart without liver	24	9.1 (5.1–17.8)	22	9.1 (5.0–16.1)
AL	Neither liver nor heart	3	4.6	2	6.3
ATTR-CM	Heart	18	9.7 (6.3–14.4)	12	9.7 (5.1–14)
**B: Liver involvement based on SAP scintigraphy**					
**Type**	**Organ involvement**	**Number of treatment-naive patients**	**Median liver stiffness in kPa (IQR)**	Number of patients in whom liver stiffness met quality criteria*	**Median liver stiffness in kPa (IQR)**
AL	Liver	41	20.9 (9.7–62.3)	36	21.9 (8.6–70.3)
AL	Heart without liver	14	9.1 (4.9–12.0)	12	9.1 (4.8–9.8)
AL	Neither liver nor heart	16	5.9 (5.0–7.5)	12	5.7 (5.0–7.5)
ATTR-CM	Heart	18	9.7 (6.3–14)	12	9.7 (5.1–14)

*IQR/M ≤ 0.30, valid measurements ≥ 10. Statistics: data are expressed median and interquartile range, from 25th to 75th percentile (IQR)

Cardiac investigations of AL amyloidosis patients with heart involvement but without liver involvement did not differ from ATTRwt-CM patients and did not differ from AL amyloidosis patients with both heart and liver involvement. In AL patients with both heart and liver involvement the left ventricular wall was less thickened and the cardiac biomarker levels higher compared to patients with ATTRwt-CM (Supplementary Table S1).

Liver stiffness and liver involvement based on consensus criteria.

**Fig. 1:**
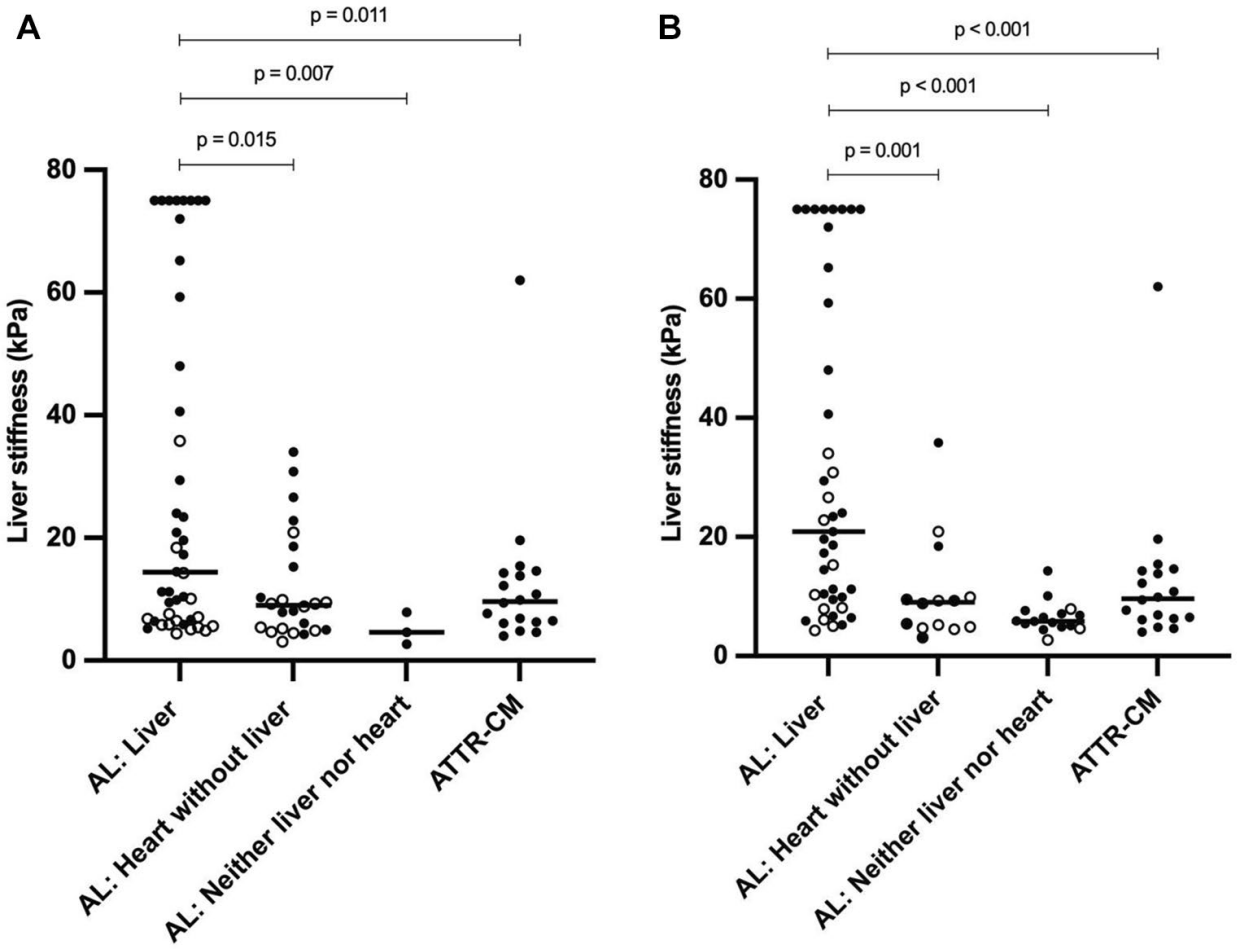
Distribution of liver stiffness among AL and ATTRwt-CM amyloidosis patients. **A**: Scatter plot showing the distribution of liver stiffness in AL amyloidosis patients with liver involvement (*N* = 44), heart involvement without liver involvement (*N* = 24) and neither liver nor heart involvement (*N* = 3) based on the consensus criteria for organ involvement, as well as wild type transthyretin amyloidosis with cardiomyopathy (ATTRwt-CM) controls (*N* = 18). Unfilled circles indicate patients in whom serum amyloid P component (SAP) scintigraphy did not indicate liver involvement. Only significant differences are shown. **B**: Scatter plot showing the distribution of liver stiffness in patients with AL amyloidosis with liver involvement (*N* = 41), heart involvement without liver involvement (*N* = 14) and neither liver nor heart involvement (*N* = 16) based on SAP scintigraphy, as well as ATTRwt-CM controls (*N* = 18). Unfilled circles indicate patients in whom consensus criteria did not indicate liver involvement. Only significant differences are shown kPa: kilopascal; LS: liver stiffness. Statistics: The Kruskal-Wallis test followed by Games-Howell post hoc analysis was used to compare liver stiffness measurements among different groups

Liver stiffness was higher in 44 AL amyloidosis patients with liver involvement compared to 24 AL amyloidosis patients with heart involvement without liver involvement, 3 AL amyloidosis patients with neither heart nor liver involvement and 18 ATTRwt-CM patients (Table 2A, Fig. 1A). The results did not change after exclusion of liver stiffness measurements that did not meet the manufacturer’s recommendations (IQR/M of ≥ 10 measurements > 0.30) (Table 2A). Ten patients had an increased liver span without elevated ALP as well as signs of cardiac failure. Because of cardiac failure they were designated as not having liver involvement.

### Liver stiffness and liver involvement based on SAP scintigraphy

Liver stiffness was higher in 41 AL amyloidosis patients with liver involvement compared to 14 AL amyloidosis patients with heart involvement without liver involvement, 16 AL amyloidosis patients with neither heart nor liver involvement and 18 ATTRwt-CM patients (Table 2B, Fig. 1B). The results did not change after exclusion of liver stiffness measurements that did not meet the manufacturer’s recommendations (IQR/M of ≥ 10 measurements > 0.30) (Table 2B).

Fifty-six patients had liver involvement based on either one or both of the standards: 29 patients based on both standards, 12 patients based on only SAP scintigraphy and 15 patients based on only consensus criteria. Twenty-seven patients were thus classified the other way round causing diversity in numbers of patients in similar categories of Table 2A and Table 2B.

Of the ten patients with an increased liver span without elevated ALP who did not have liver involvement based on the consensus standard because of cardiac failure (see above) three did have liver involvement based on liver uptake on SAP scintigraphy. Liver stiffness was increased in two of the three patients and in one of the seven remaining patients.

### Univariate and multivariate linear regression analysis for liver stiffness

In patients with AL amyloidosis fairly strong correlations were found between liver stiffness and both GGT (r_s_= 0.740 *p* < 0.001) and hepatic tracer uptake on SAP scintigraphy (expressed as LBR) (r_s_ = 0.740, *p* < 0.001) (Supplementary Figure S3A). Moderate correlations were found between liver stiffness and both ALP (r_s_ = 0.672, *p* < 0.001) and ALT (r_s_ = 0.598, *p* < 0.001) (Supplementary Figure S3B). Weak correlations were found between liver stiffness and liver span (r_s_ = 0.385, *p* < 0.001) (Supplementary Figure S3C), NT-proBNP (r_s_ = 0.268, *p* = 0.024), Troponin T (r_s_ = 0.393 *p* = 0.001), and creatinine (r_s_ = 0.297, *p* = 0.012). No correlations were found between liver stiffness and age, BMI and proteinuria.

All variables that significantly correlated with liver stiffness in univariate analysis were included in multivariate linear regression analysis except for GGT and Troponin T as these variables are interrelated to ALP and NT-proBNP, and not part of the criteria for organ response and progression [30], respectively. Multivariate linear regression showed that only ALP, hepatic tracer uptake on SAP scintigraphy (expressed as LBR) and creatinine were independently related to liver stiffness (Table 3).

**Table 3:** Univariate and multivariate linear regression analysis for liver stiffness in AL amyloidosis patients

	Univariate Analysis (Spearman’s rho)		Multivariate Analysis (*R*^2^ = 0.78)	
Variable	*r*	*p*-value	Beta (95% CI)	*p*-value
Age	-0.012	NS		
BMI	0.092	NS		
**ALT**	**0.598**	**< 0.001**	0.083 (-0.072 to 0.240)	NS
**ALP**	**0.672**	**< 0.001**	0.454 (0.295 to 0.602)	< 0.001
GGT	0.740	< 0.001		
**Liver span**	**0.385**	**< 0.001**	0.099 (-0.035 to 0.233)	NS
**NT-proBNP**	**0.268**	**0.024**	-0.041 (0.000 to 0.000)	NS
Troponin T	0.393	< 0.001		
**Creatinine**	**0.297**	**0.012**	0.172 (0.046 to 0.302)	0.009
Proteinuria	0.033	NS		
**LBR**	**0.740**	**< 0.001**	0.483 (0.349 to 0.617)	< 0.001

ALT: alanine aminotransferase; ALP: alkaline phosphatase; GGT: gamma-glutamyl transferase; NT-proBNP: N-terminal fragment of pro brain natriuretic peptide; LBR: liver to blood ratio; 95% CI: 95% confidence interval. In bold are the variables that were included in multivariate analysis. Statistics: Univariate analysis: Spearman’s correlations coefficient (rs); multivariate analysis with enter inclusion of variables was performed

### Diagnostic accuracy of liver stiffness, ALP, liver span and SAP scintigraphy

**Fig. 2:**
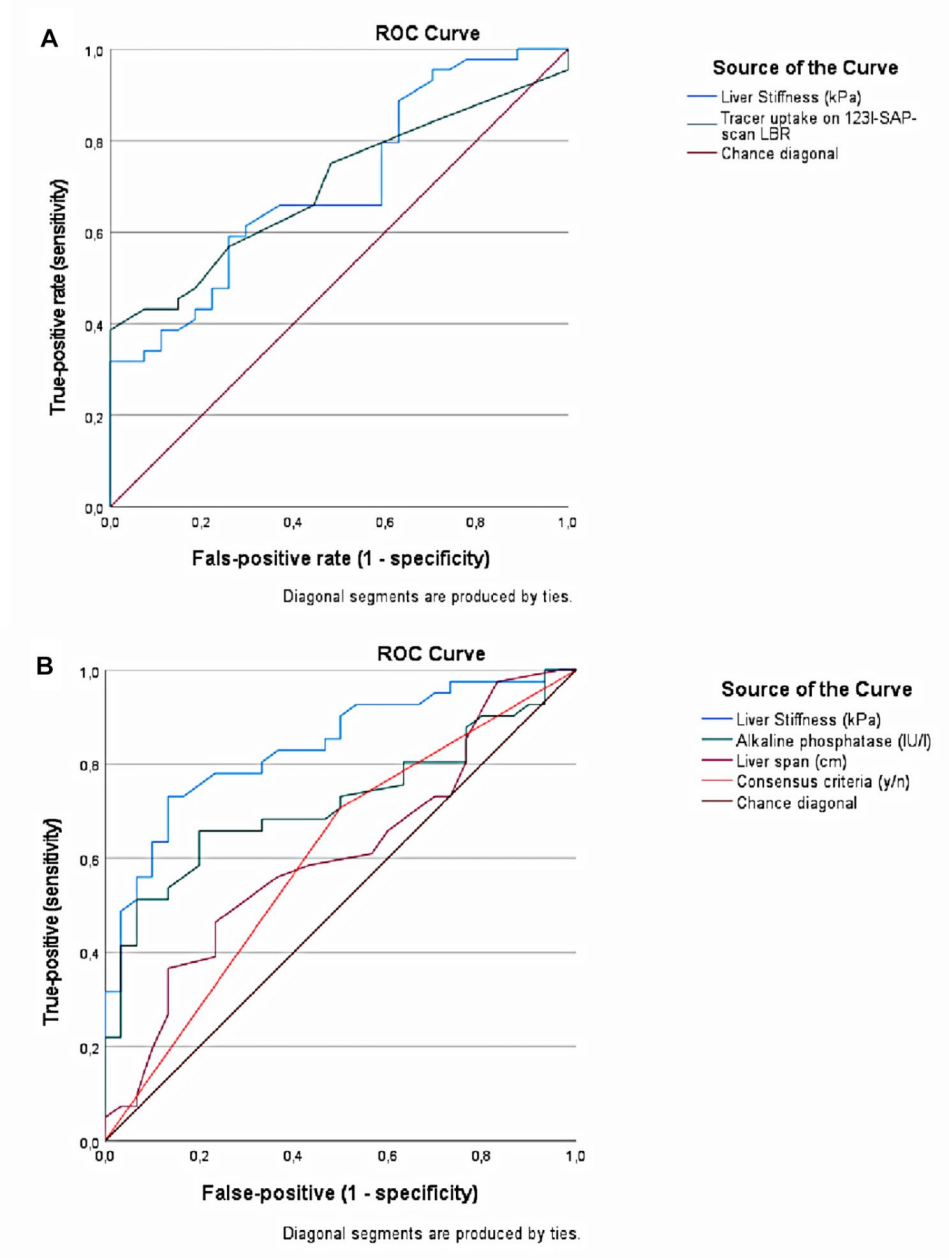
Diagnostic accuracy of liver stiffness. **A**: Receiver operating characteristic (ROC) curves in treatment-naïve AL amyloidosis patients comparing liver stiffness and liver tracer uptake (LBR) on serum amyloid P component (SAP) scintigraphy using consensus criteria as standard for liver involvement. **B**: ROC curves in treatment-naïve AL amyloidosis patients comparing liver stiffness, alkaline phosphatase, liver span and consensus criteria using SAP scintigraphy as standard for liver involvement

When the consensus criteria were used as standard for establishing liver involvement, liver stiffness had moderate diagnostic accuracy (ROC AUC liver stiffness = 0.702; 95% CI: 0.580–0.824). The diagnostic accuracy of liver stiffness was comparable to the diagnostic accuracy of liver tracer uptake on [^123^I]-SAP scan (LBR) (ROC LBR = 0.701; 95% CI: 0.582–0.820) (Fig. 2A). When taking into account that normal liver stiffness values are below 6 kPa, that median liver stiffness in patients with decompensated heart failure is 8.8 kPa [31], and that liver stiffness above 12.5 kPa is indicative for severe fibrosis in different chronic liver diseases [32], a cut-off value of 14.4 kPa was chosen with an associated sensitivity figure of 50% and specificity of 74%.

When SAP scintigraphy was used as standard for establishing liver involvement, liver stiffness had good diagnostic accuracy (ROC AUC liver stiffness = 0.837; 95% CI: 0.745–0.930) providing a sensitivity figure of 63% and specificity of 90% when 14.4 kPa was used as cut-off value. Compared to ALP, liver span, and the consensus criteria, liver stiffness had the highest diagnostic accuracy followed by ALP (ROC AUC ALP: 0.712; 95% CI: 0.591–0.833), liver span (ROC AUC liver span = 0.608; 95% CI: 0.475–0.741), and consensus criteria (ROC AUC consensus criteria = 0.604; 95% CI 0.469–0.738) (Fig. 2B). The combination of liver stiffness > 14.4 kPa and/or ALP > 1.5x ULN further increased the diagnostic accuracy for liver involvement based on SAP scintigraphy providing a sensitivity figure of 68% and specificity of 90% (Table S2).

### Longitudinal analysis

Liver stiffness measurements were obtained in 8 AL amyloidosis patients with liver involvement (as determined by SAP scintigraphy) before the start of treatment and after a median of 12 months (range 6–17) of treatment. Of the 7 patients with a complete or very good partial response, the median liver stiffness decreased from 22.8 kPa to 15.2 kPa (*p* = 0.03). In one patient with a partial response, liver stiffness was 5.2 kPa before the start of treatment and 6.2 kPa 12 months after the start of treatment.

## Discussion

Measuring liver stiffness is useful for establishing liver involvement in patients with AL amyloidosis. When liver involvement is based on the consensus criteria, the diagnostic accuracy of both liver stiffness and SAP scintigraphy are moderate. However, when liver involvement is based on SAP scintigraphy, liver stiffness has good diagnostic accuracy and performs better in comparison to ALP and liver span individually as well as to their combination in the consensus criteria. Using SAP scintigraphy as standard, the combination of liver stiffness > 14.4 kPa or ALP > 1.5 x ULN carries moderate sensitivity (68%) and good specificity (90%).

Liver stiffness in AL amyloidosis patients with liver involvement was increased based on both the consensus criteria and SAP scintigraphy. The current findings are consistent with two previous studies [8, 33]. The first study involving 18 apolipoprotein A-I (AApoA-I) amyloidosis patients with liver involvement found an average liver stiffness of 21.9 (± 18.3) kPa compared to 6.05 (± 4.6) kPa in the 76 AApoA-I amyloidosis patients without liver involvement [33]. The second study, by Loustaud-Ratti et al., found a median liver stiffness of 27.4 (range 10.3–75) kPa in 11 AL amyloidosis patients with liver involvement [8]. The second study also investigated a cut-off value, based on a ROC curve, for differentiating the 11 AL amyloidosis patients from a control group without amyloidosis including: negative controls, patients with hepatitis C, and patients with right sided heart failure. They found an optimal cut-off value of 10.1 kPa, obtaining a sensitivity figure of 90.9% and specificity of 70.9%. However, in order to diagnose in practice highly significant amyloid infiltration of the liver, prioritizing high specificity, a higher cut-off value of 17.3 kPa was chosen, obtaining a sensitivity figure of 63.6% and specificity of 92.4%. In the current study an optimal cut-off value of 14.4 kPa was obtained with comparable sensitivity and specificity. The figures for sensitivity and specificity were 50% and 74%, respectively, using the consensus criteria as standard and 63% and 90%, respectively, using the SAP scintigraphy as standard. Taking into account that normal liver stiffness values are below 6 kPa, that median liver stiffness in patients with decompensated heart failure is 8.8 kPa [31], and that liver stiffness above 12.5 kPa is indicative for severe fibrosis in different chronic liver diseases [32], the optimal cut-off value with high specificity will probably lie somewhere between 14.4 and 17.3 kPa. Larger studies are needed to further investigate the best cut-off value.

Since liver stiffness may be affected by heart failure [31], we also studied liver stiffness in AL amyloidosis patients with heart involvement but without liver involvement and in ATTRwt amyloidosis patients with cardiomyopathy. Liver stiffness was significantly higher in AL amyloidosis patients with liver involvement compared to patients with heart involvement without liver involvement or ATTRwt-CM amyloidosis patients. Although a weak positive correlation was found between liver stiffness and NT-proBNP, the effect of heart failure on liver stiffness seems insignificant compared to the effect of amyloid deposition in the liver as NT-proBNP was no longer related to liver stiffness in multivariate analysis.

As expected, ALP and liver tracer uptake on SAP scintigraphy were independently related to liver stiffness in multivariate analysis. Unexpectedly, liver span was not independently related to liver stiffness. Congestive heart failure may increase liver span [10] and therefore could bias the interpretation of liver span as reflection of liver involvement. For this reason, the consensus criteria for liver involvement state that only in the absence of heart failure a total liver span > 15 cm implicates liver involvement. In routine clinical practice this criterion is not always easy to work with, because a considerable number of patients have both heart and liver involvement and it is sometimes difficult to decide whether fluid overload is caused by heart failure, nephrotic syndrome, or both. Another unexpected finding was that liver stiffness was independently related to the serum creatinine. Since AL amyloidosis patients with liver involvement mostly have multi-organ involvement [34], we first sought our explanation in concomitant kidney involvement. However, the distribution kidney involvement was similar between patients with and patients without liver involvement (21 of the 44 with consensus criteria; 22 of the 41 with SAP scintigraphy). Though serum creatinine was significantly higher in patients with liver involvement compared to patients without liver involvement based on SAP scintigraphy, this was not seen in patients with liver involvement based in the consensus criteria (data not shown). Thus, a clarification of the relation between creatinine and liver stiffness is still lacking.

The manufacturer of the Fibroscan recommends caution with the interpretation of results of < 10 valid measurements and/or IQR/M > 0.30 [7]. In the current study, one patient had 5 valid measurements with an IQR/M of 0.89, in another 16 patients the IQR/M was > 0.30 (10 patients had an IQR/M below 0.50 and 6 above 0.50). The proportion of measurements that did not meet the manufacturer’s recommendations (19%) was slightly higher compared to that found in the largest transient elastography series reported to date (16%) [35]. The report showed that obesity or limited operator experience can cause unreliable results. We did not find a relation between BMI and quality of the measurements (data not shown) but we do recognize that measurement can be difficult in obese patients. Initially, we did not repeat measurements if the IQR/M was > 0.30, but during the course of the study we decided to repeat measurements in case of IQR/M > 0.30 and it proved possible to obtain results that met the manufacturer’s recommendations in most cases. We did not find a difference between results that did or did not meet the recommendations. Another study in a large population (*n* = 1165) of patients with chronic liver disease showed that liver stiffness results that do meet the recommendations are not more accurate for the diagnosis of liver cirrhosis than results that do not meet these recommendations [7]. As we wanted this study to reflect the diagnostic accuracy of liver stiffness in a real-life setting, we included all results. Reassuringly, exclusion of results that did not meet the manufacturer’s recommendations did not change the outcomes.

Liver biopsy is considered the gold standard, but given the risk of the procedure, especially in patients with systemic amyloidosis, it is unlikely that a validation study against liver biopsies will ever be performed. The non-invasive consensus criteria for liver involvement are only meant to implicate liver involvement and have not been validated against the liver biopsy. There are good arguments to challenge the selection of liver span as one of the two key elements of the consensus criteria. Difficulties of interpreting liver span encountered in routine practice [21], the mere finding that 25% of more than two thousand healthy subjects had a liver span above 15 cm [36], the futility of liver span in case of cardiac failure and its disappointing diagnostic accuracy (ROC AUC 0.609) based on SAP scintigraphy as standard (Fig. 2B) all indicate that liver span fails to fulfil its key role. Both ALP and liver span are not specific for amyloidosis and are increased in a number of diseases, whereas SAP scintigraphy has been designed to be specific for amyloidosis. The availability of SAP scintigraphy to establish liver involvement may therefore be considered a strength of the current study. Liver stiffness has moderate diagnostic accuracy (AUC 0.702) when the consensus criteria are considered the standard and even good diagnostic accuracy (AUC 0.837) when SAP scintigraphy is considered the standard for liver involvement. As liver stiffness decreased in patients with a complete or very good partial response, it may also be useful for assessing organ response. The hematologic response criteria [37] and the cardiac organ response criteria [38] have been updated recently. Time may have come to consider also an update of the non-invasive consensus criteria for liver involvement. Larger studies are needed to investigate whether replacing liver span with liver stiffness might indeed improve the diagnostic accuracy of the consensus criteria.

As discussed above, the lack of a true gold standard for liver involvement is the main limitation of this study and another limitation is the wide range of measurements of liver stiffness in a number of individuals. Although this study is the largest study so far investigating the diagnostic value of liver stiffness in AL amyloidosis, the subgroups of patients are small. Selection bias of the study population into the direction of liver involvement is not a limitation, because it does not affect the findings of the current study focused on diagnostic performance of liver stiffness. However, to learn the actual diagnostic performance of liver stiffness in the general population of patients with AL amyloidosis, and before the found cut-offs for liver stiffness can be recommended, a validation study should be performed in another cohort consisting of randomly selected patients.

In conclusion, this study shows that measurement of liver stiffness using transient elastography is useful in clinical practice to establish liver involvement in patients with AL amyloidosis. It has moderate or good diagnostic accuracy depending on what is considered the substitute of the gold standard for liver involvement. Liver stiffness with a cut-off value of 14.4 kPa might be a good candidate to replace liver span within the current consensus criteria for liver involvement. The prognostic value of liver stiffness and the value of liver stiffness in monitoring organ response during treatment is currently being evaluated.

## Electronic supplementary material

Below is the link to the electronic supplementary material.

Supplementary Material 1

Supplementary Material 2

## Data Availability

Data AvailabilityAny inquiries regarding supporting data availability of this study should be directed to the corresponding author.

## Abbreviations

AL Amyloidosis: immunoglobulin light chain amyloidosis
ApoA-I Amyloidosis: Apolipoprotein A-I amyloidosis
ATTRwt: wild type transthyretin amyloidosis
ATTRwt-CM: wild type transthyretin amyloidosis with cardiomyopathy
ALP: Alkaline phosphatase
ALT: Alanine aminotransferase
BMI: Body mass index
CT scan: Computed tomographic imaging
GGT: Gamma-glutamyl transferase
kPa: kilopascal
[^123^I]-SAP: with iodine-123 labeled serum amyloid P component
IQR/M: Interquartile range/median ratio
LBR: Liver to blood pool ratio
LS: liver stiffness
NT-proBNP: N-terminal pro B-type natriuretic peptide
ROC curve: Receiver operating characteristic curve
