# Supplementary material for: Diagnostic performance of liver stiffness as marker of liver involvement in systemic immunoglobulin light chain (AL) amyloidosis
Source: Ann Hematol. 2024 Aug 16;104(1):653–63. doi: 10.1007/s00277-024-05932-4 (PMC11868187; doi:10.1007/s00277-024-05932-4)
Supplement: Supplementary file 1 — Supplementary Material 1 [file 277_2024_5932_MOESM1_ESM.pdf]

Supplemental figures

Article title: Diagnostic performance of liver stiffness as marker of liver involvement in systemic immunoglobulin light chain (AL) amyloidosis

Journal name: Annals of Hematology

Authors: Anne F. Brunger, Hendrea S.A. Tingen, Johan Bijzet, Ronald van Rheenen, Hans Blokzijl, Wilfried W.H. Roeloffzen, Ewout J. Houwerzijl, Friso L.H. Muntinghe, Riemer H.J.A. Slart, Reinold O.B. Gans, Bouke P.C. Hazenberg, Hans L.A. Nienhuis

Affiliation corresponding author: Department of Rheumatology & Clinical Immunology, University Medical Center Groningen

E-mail address corresponding author: annefloorbrunger@hotmail.com

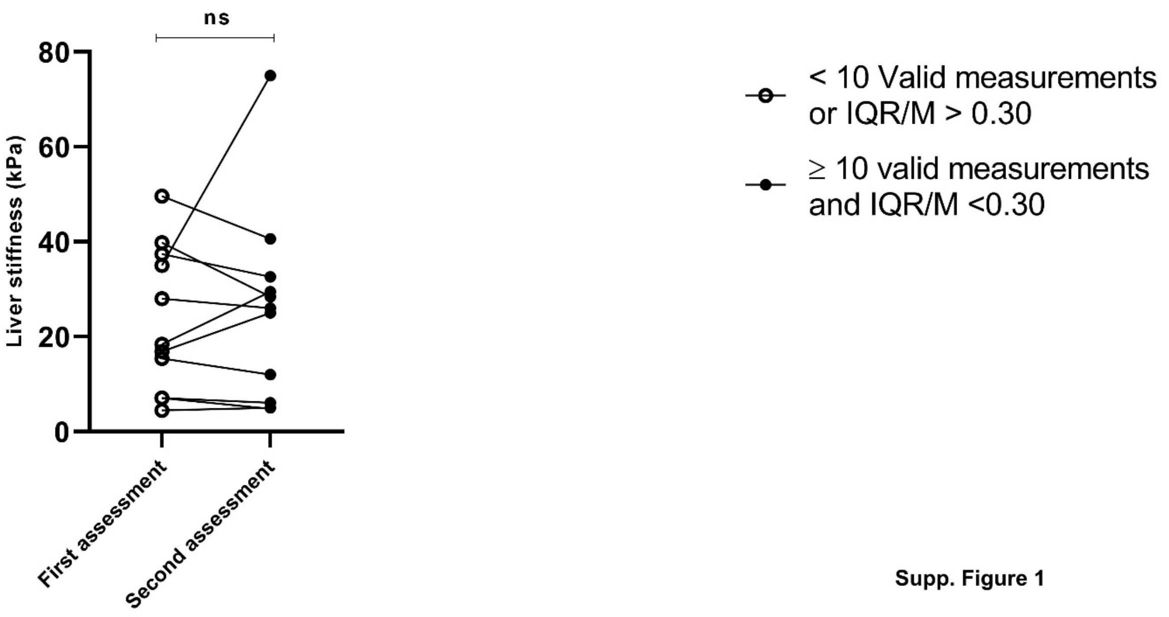

Fig. S1 Liver stiffness in 11 patients in whom liver stiffness was assessed for a second time because the result of the first measurements did not meet the recommendations of >10 valid measurements or IQR/M <0.30

A Liver involvement based on consensus criteria

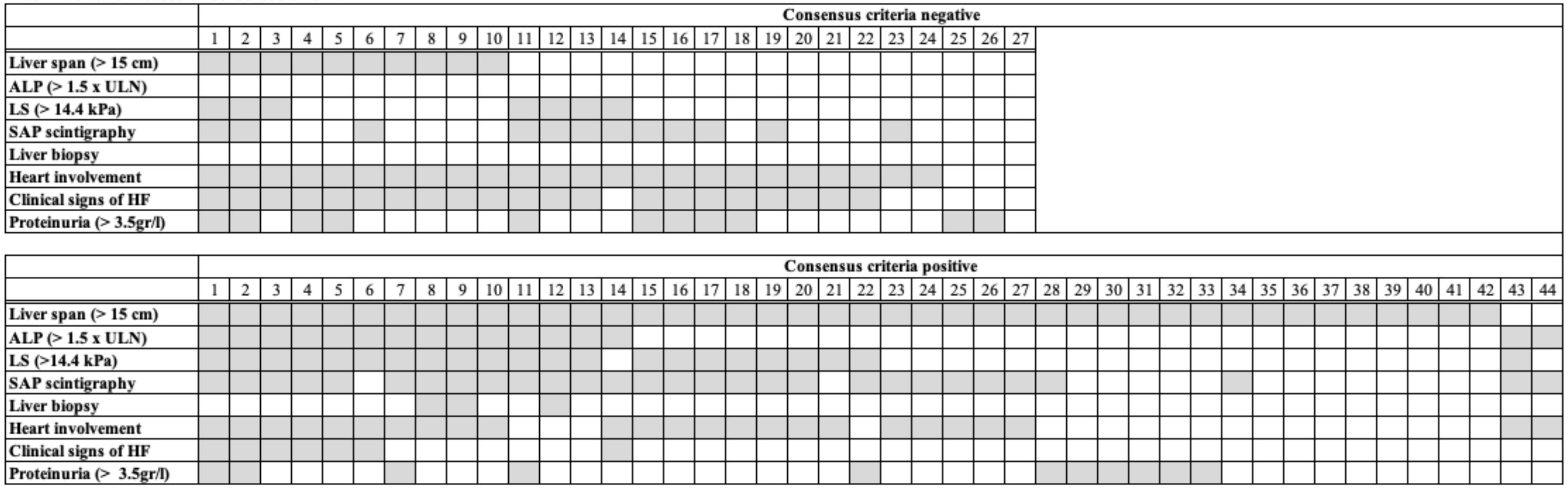

B Liver involvement based on SAP scintigraphy

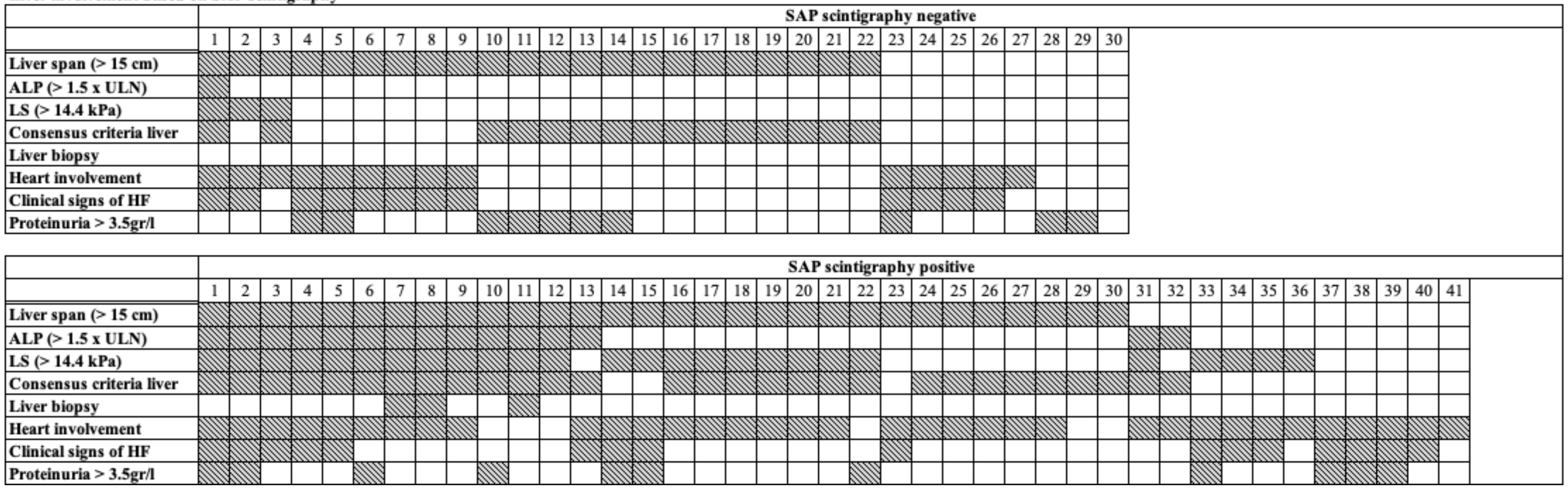

Fig. S2 Results of markers of liver, heart involvement and proteinuria based on either the consensus criteria for liver involvement (A) or SAP scintigraphy (B)

The solid grey fields indicate a positive result in an individual patient classified according to the consensus criteria and the grey shaded fields indicate a positive result in an individual patient classified according to the SAP scintigraphy. A positive test result was defined as: liver span >15 cm, ALP > 1.5 times the upper limit off the institutional normal value (>147 IU/l for woman and >172 IU/l for men), liver stiffness > 14.4 kPa, heart involvement according to Gertz et al [10], clinical signs of heart failure: increased central venous pressure, peripheral oedema or basal crepitation in the lungs in a patient with cardiac involvement, proteinuria > 3.5gr protein in urine per day, consensus criteria for liver involvement according to Gertz et al [10]. ALP: alkaline phosphatase; ULN: upper limit of normal; LS: liver stiffness; HF: heart failure; SAP: serum amyloid P component.

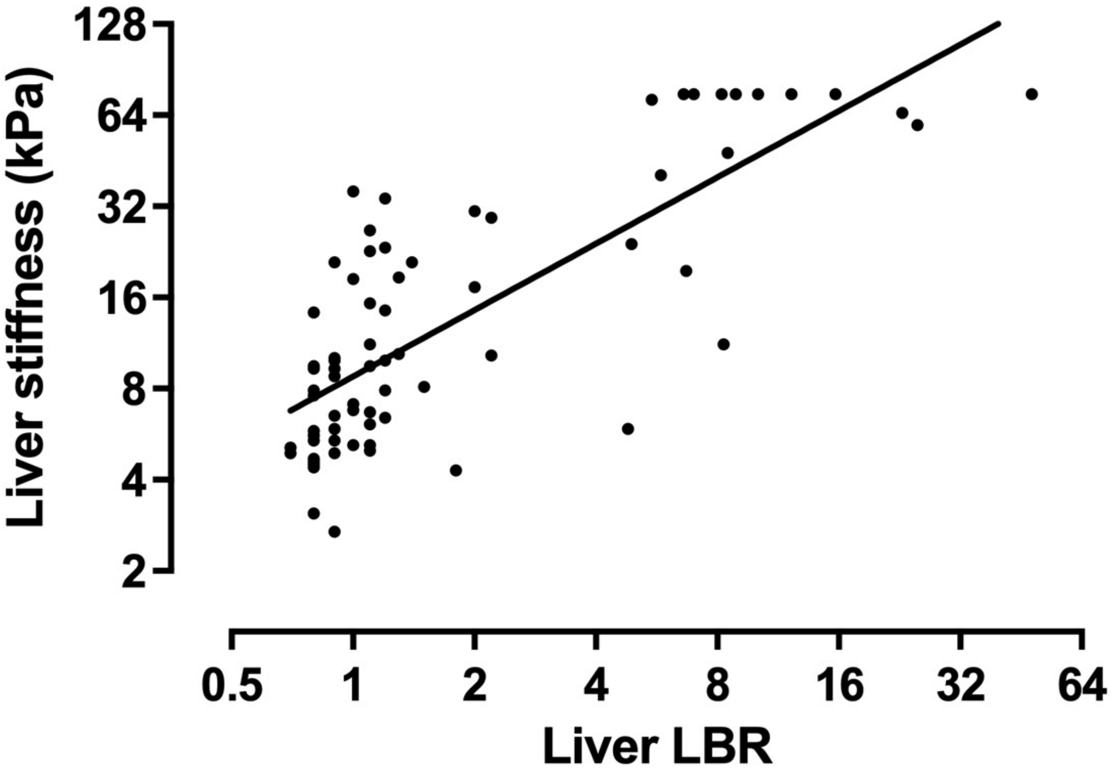

Fig. S3A Liver stiffness and hepatic tracer uptake on SAP scintigraphy (LBR)

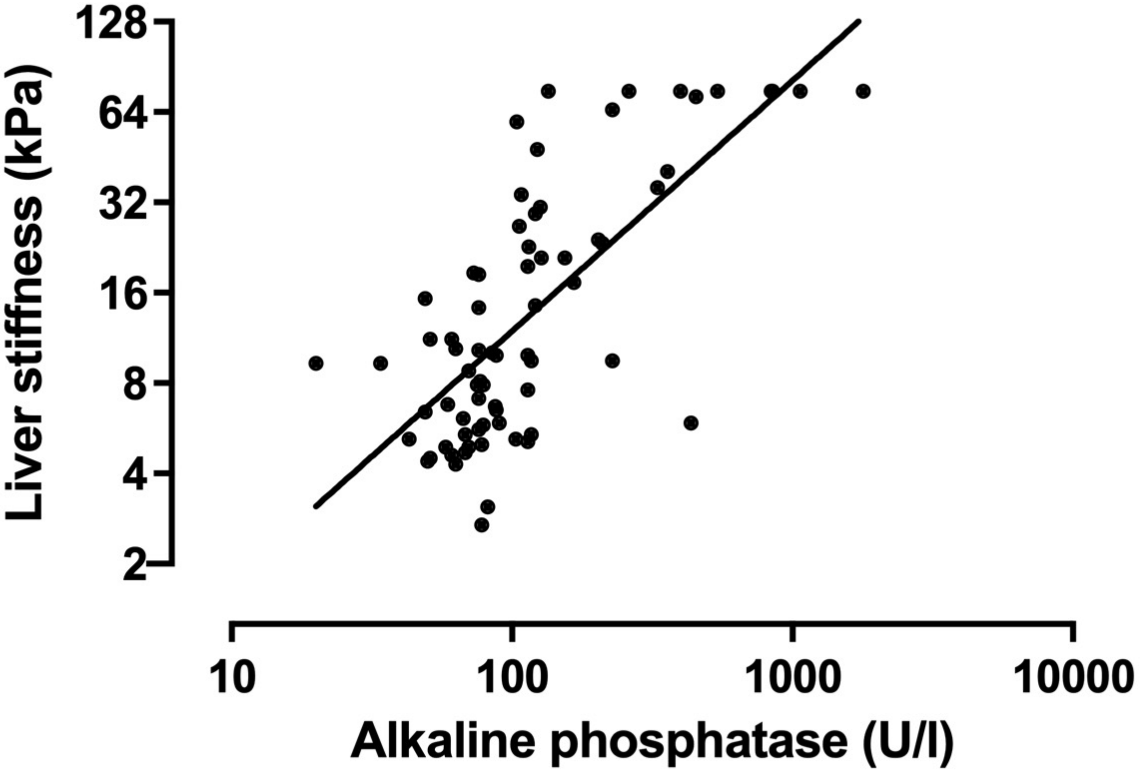

Fig. S3B Liver stiffness and alkaline phosphatase (ALP)

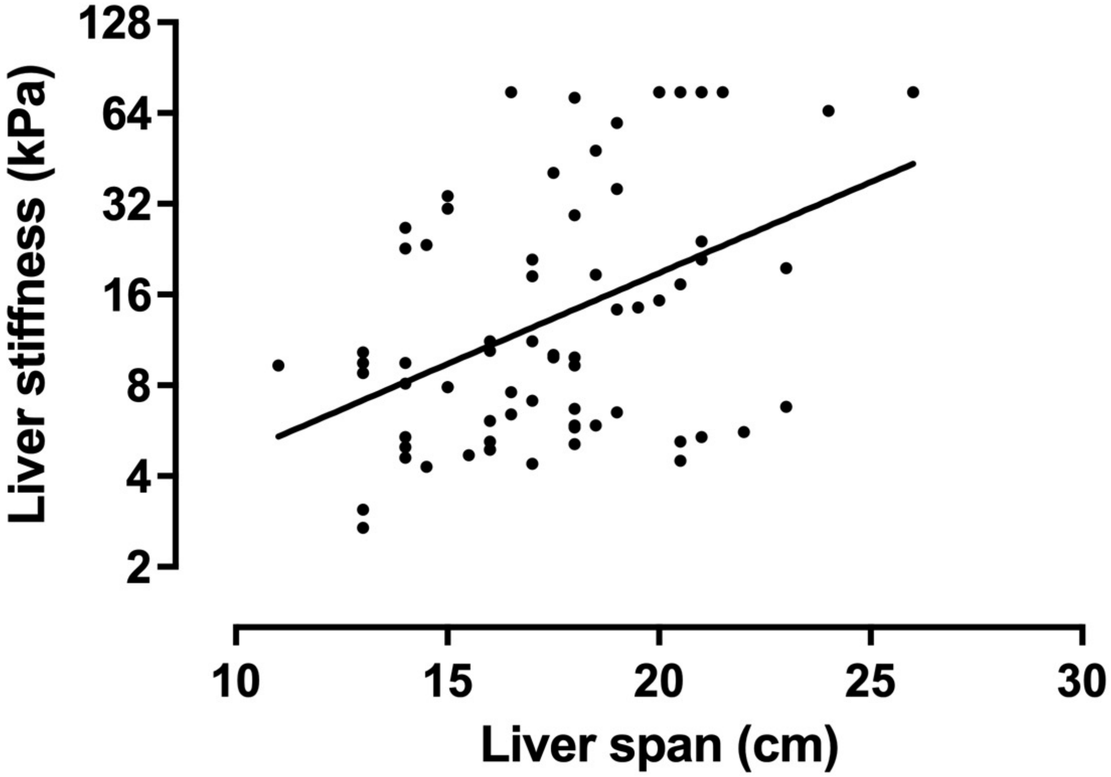

Fig. S3C Liver stiffness and liver span
