# Supplementary material for: Diagnostic performance of liver stiffness as marker of liver involvement in systemic immunoglobulin light chain (AL) amyloidosis
Source: Ann Hematol. 2024 Aug 16;104(1):653–63. doi: 10.1007/s00277-024-05932-4 (PMC11868187; doi:10.1007/s00277-024-05932-4)
Supplement: Supplementary file 2 — Supplementary Material 2 [file 277_2024_5932_MOESM2_ESM.pdf]

Supplemental tables

Article title: Diagnostic performance of liver stiffness as marker of liver involvement in systemic immunoglobulin light chain (AL) amyloidosis

Journal name: Annals of Hematology

Authors: Anne F. Brunger, Hendrea S.A. Tingen, Johan Bijzet, Ronald van Rheenen, Hans Blokzijl, Wilfried W.H. Roeloffzen, Ewout J. Houwerzijl, Friso L.H. Muntinghe, Riemer H.J.A. Slart, Reinold O.B. Gans, Bouke P.C. Hazenberg, Hans L.A. Nienhuis

Affiliation corresponding author: Department of Rheumatology & Clinical Immunology, University Medical Center Groningen

E-mail address corresponding author: annefloorbrunger@hotmail.com

Table S1. Cardiac investigations of AL amyloidosis patients with heart involvement with and without liver involvement (according to consensus criteria or SAP scintigraphy) and of ATTRwt amyloid cardiomyopathy patients.

	**Consensus criteria**			
	**AL: Heart and liver Median (range)**	**AL: Heart without liver Median (range)**	**ATTRwt Median (range)**	**p-value**
**NT-proBNP (ng/L)**	4028 (1651 - 8450) (N = 25)	2638 (1327 - 19396) (N = 24)	2395 (210 – 13918) (N = 18)	n.s.
**Troponine T (ng/L)**	87 (34 - 155) (N = 25)	64 (42 - 109) (N = 24)	65 (23 -223) (N =18)	n.s.
**Mean left ventricular wall thickness (mm)**	15 (12 – 17) (N = 12)	15 (12 – 16.0) (N = 9)	18 (12 -22) (N = 8)	0.018[a]
	**SAP scintigraphy**			
	**AL: Heart and liver Median (range)**	**AL: Heart without liver Median (range)**	**ATTRwt Median (range)**	**p-value**
**NT-proBNP (ng/L)**	4028 (1972 - 11143) (N = 35)	2118 (587 - 9680) (N = 14)	2395 (210 – 13918) (N = 18)	<0.001[a]
**Troponine T (ng/L)**	79 (37 - 119) (N = 35)	60 (25 - 126) (N = 14)	65 (23 -223) (N =18)	<0.001[a]
**Mean left ventricular wall thickness (mm)**	15 (12 – 17) (N = 18)	15 (12 – 16) (N = 9)	18 (12 -22) (N = 8)	<0.001[a]

AL: AL amyloidosis patients; NT-proBNP, N-terminal pro B-type natriuretic peptide.
a: significant difference between AL: Heart and liver and ATTRwt groups; n.s.: no significant differences among the groups
Statistics: Kruskal-Wallis test followed by the Games-Howell post hoc analysis.

Table S2. Sensitivity and specificity of different criteria for liver involvement

	Liver involvement based on consensus criteria	
**Criterium**	**Sensitivity**	**Specificity**
LS (> 14.4 kPa)	50%	74%
SAP scintigraphy	63%	90%
	**Liver involvement based on SAP scintigraphy**	
	**Sensitivity**	**Specificity**
ALP (>1.5x ULN)	37%	97%
Liver span (> 15cm)	73%	27%
Consensus criteria	71%	50%
LS (>14.4 kPa)	63%	90%
LS (>14.4 kPa) or ALP (>1.5x ULN)	68%	90%
LS (>14.4 kPa) or Liver span (> 15cm)	85%	27%

LS: liver stiffness; ALP: alkaline phosphatase; ULN: upper limit of normal; SAP: serum amyloid P component.

Statistics: Sensitivity and specificity calculations.
